# Three-Dimensional Visualization With Virtual Reality Facilitates Complex Live Donor Renal Transplant

**DOI:** 10.31486/toj.22.0008

**Published:** 2022

**Authors:** Dennis Sonnier, W. Peter Sawyer, John Seal, Colin Curtis, Jack McGee, Alec Slayden, Korak Sarkar

**Affiliations:** ^1^ Section of Transplant Surgery, Ochsner Clinic Foundation, New Orleans, LA; ^2^ The University of Queensland Medical School, Ochsner Clinical School, New Orleans, LA; ^3^ Department of General Surgery, Ochsner Clinic Foundation, New Orleans, LA; ^4^ Medical 3D Lab, Ochsner Clinic Foundation, New Orleans, LA; ^5^ Department of Neurology, Ochsner Clinic Foundation, New Orleans, LA

**Keywords:** *Kidney transplantation*, *living donor*, *radiology*, *virtual reality*

## Abstract

**Background:** Living donor renal transplant involves highly technical operations in both a healthy donor and a recipient with end-stage kidney disease. Contrast-enhanced computed tomography angiography (CTA) is used to assess critical donor anatomy, but its interpretation becomes increasingly difficult as renal anatomy becomes more complex.

**Case Report:** A related donor was denied because of prohibitive anatomy seen on the pretransplant evaluation CTA. As the donor was highly motivated to donate, CTA DICOM images were segmented to create a 3-dimensional (3D) model that could be evaluated in an immersive and stereoscopic virtual reality (VR) environment. The donor's anatomy was found to be acceptable, and he was approved.

**Conclusion:** In live donor nephrectomy candidates, 3D reconstruction and VR visualization can be used to facilitate appreciation of complex anatomy.

## INTRODUCTION

Live donor renal transplant (LDRT), the transplantation of a kidney procured from a living donor, involves invasive and highly technical operations in both the healthy donor and the recipient with end-stage kidney disease. Long wait times and poor prognosis with long-term dialysis^1^ make LDRT a life-saving as well as a life-improving therapy. Aggressive utilization of LDRT is imperative to today's kidney transplant practice, requiring pushing boundaries on many criteria such as donor age, body mass index, HIV status, immunologic compatibility, and kidney donor anatomy.^2-4^

Renal vascular anatomy and kidney size are important considerations when approving live donors and when selecting the appropriate kidney laterality for laparoscopic procurement. Obtaining detailed data regarding renal artery and vein number, length, diameter, and position is critical to planning donor nephrectomy. Relative kidney size, determined by split volume and/or split function, is important to both donor and recipient renal function postprocedure.^5^

Utilizing every available tool or technology leads to improved understanding of anatomic details. Accurate assessment of these anatomic details is paramount to planning donor and recipient operations.

We present a case in which 3-dimensional (3D) visualization with virtual reality (VR) facilitated decision-making in approving a living donor and in selecting the appropriate kidney for transplantation.

## CASE REPORT

The donor candidate was a healthy 35-year-old male wanting to donate a kidney to his brother who had been on hemodialysis for 3 years because of focal segmental glomerulosclerosis (FSGS). He underwent standard donor evaluation at our center, including consultation with multidisciplinary transplant specialists (nephrology, surgery, infectious diseases, nutritionist, and social worker), extensive blood and urine testing, and multiphase thin-slice computed tomography angiography (CTA), and he was assigned a donor advocate. The donor had a body mass index of 23.6 kg/m^2^, normal blood pressure, no preexisting medical problems, and a history of laparoscopic hernia repair, laparoscopic appendectomy, and jaw surgery.

The selection committee initially declined the donor because of the high vascular complexity of the bilateral kidneys as they appeared on 2-dimensional (2D) CTA images in the axial and coronal views. The right kidney had 2 arteries originating 1.2 cm apart on the aorta. Both arteries appeared to be bifurcating posterior to the inferior vena cava (IVC). The right renal vein was only 1.5 cm long.

The left kidney had 2 arteries originating 0.5 cm apart. One artery had an early bifurcation 1.6 cm from the aorta. Additionally, the arteries crossed, and the point of crossing coincided with the branching points, making it difficult to distinguish between branching and crossing and raising the concern that 3 or even 4 renal arteries would require reconstruction. The left renal vein was 6 cm long.

**Figure 1.**
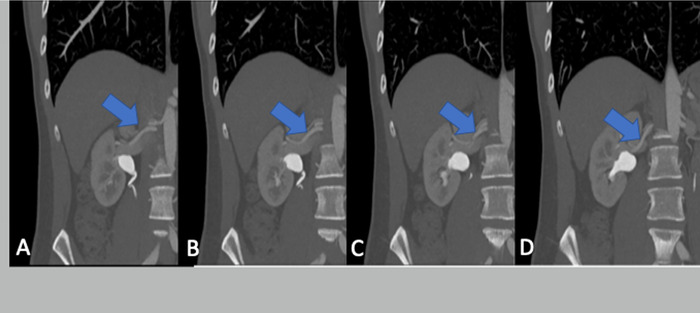
Coronal computed tomography angiography images of the donor's right kidney demonstrate complex extrarenal bifurcation (posterior to the inferior vena cava), crossing of 2 main arteries, and a short renal vein. Arrows point to the arteries. From left to right (A to D), the images become more posterior.

**Figure 2.**
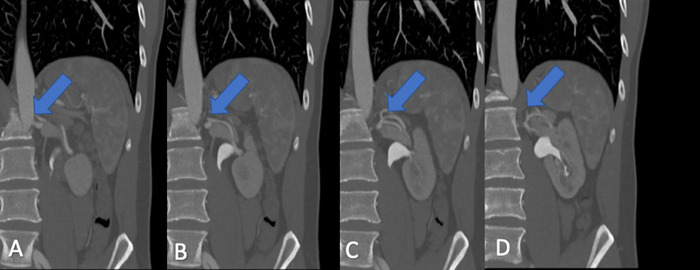
Coronal computed tomography angiography images of the donor's left kidney demonstrate complex extrarenal bifurcation and crossing of 2 main arteries, highlighting the ambiguity regarding the number of arteries potentially present after donor nephrectomy. Arrows point to the arteries. From left to right (A to D), the images become more posterior.

Additionally, gross measurements on the CTA demonstrated a significant size difference in the organs, with the left kidney being larger than the right. Discrete volume measurements were not available with the 2D CTA images. The multiple arteries and short vein on the right kidney (Figure 1) were deemed prohibitive, as was the possibility for 3 or 4 arteries on the left kidney (Figure 2).

The patient was highly motivated to donate, so we decided to reconsider his candidacy with the aid of 3D visualization in VR to understand his renal vascular anatomy more precisely.

**Figure 3.**
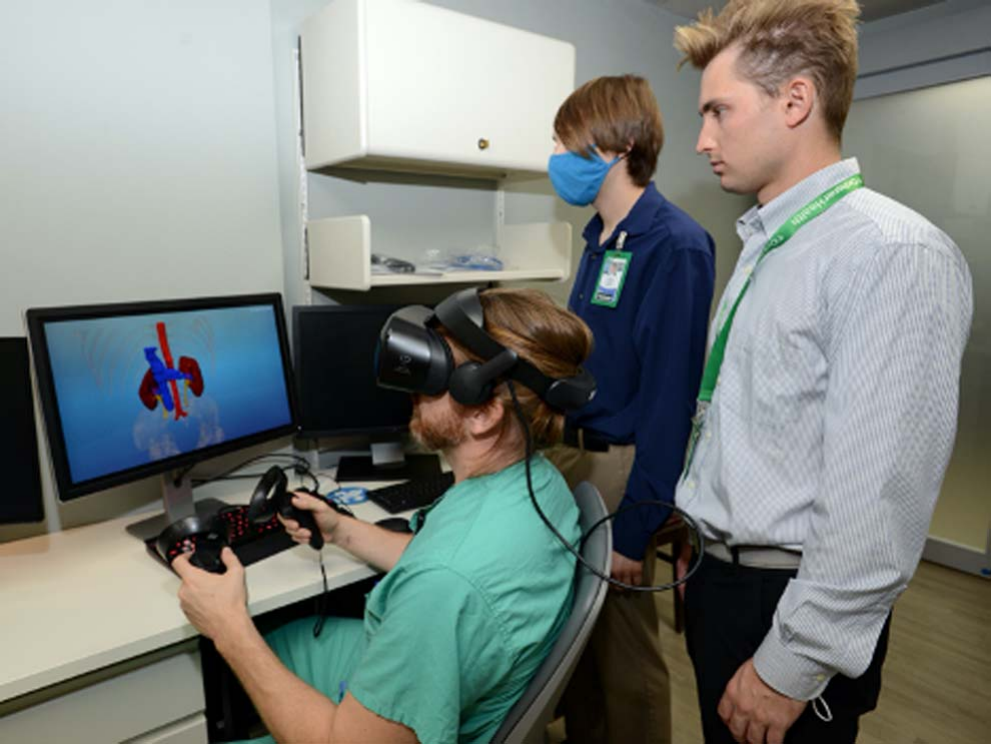
The surgeon and segmentation team evaluate a 3-dimensional model in virtual reality using the Samsung HMD Odyssey headset and hand controls that facilitate manipulation of the model and the immersive quality of the virtual reality.

CTA imaging of the abdomen and pelvis with contrast was obtained and deidentified in a standard process in conjunction with the Ochsner Radiology Department. 3D Slicer,^6^ an open-source software, was used to segment DICOM data and create a 3D stereolithography (STL) object. Multiple transplant surgeons viewed the STL in VR using an internally developed application and a commercially available head-mounted display (Samsung HMD Odyssey) (Figure 3).

VR allows for stereoscopic visualization and delineation of complex anatomy that is not possible with traditional 2D imaging modalities. The resulting volumetric data suggested only a slight difference in kidney volume measurements (53% left and 47% right) that allowed for either kidney to be potentially acceptable for donation from a size and function standpoint.

As expected, the right kidney had a prohibitively short vein and 2 arteries crossing posterior to the IVC, complicating the approach.

**Figure 4.**
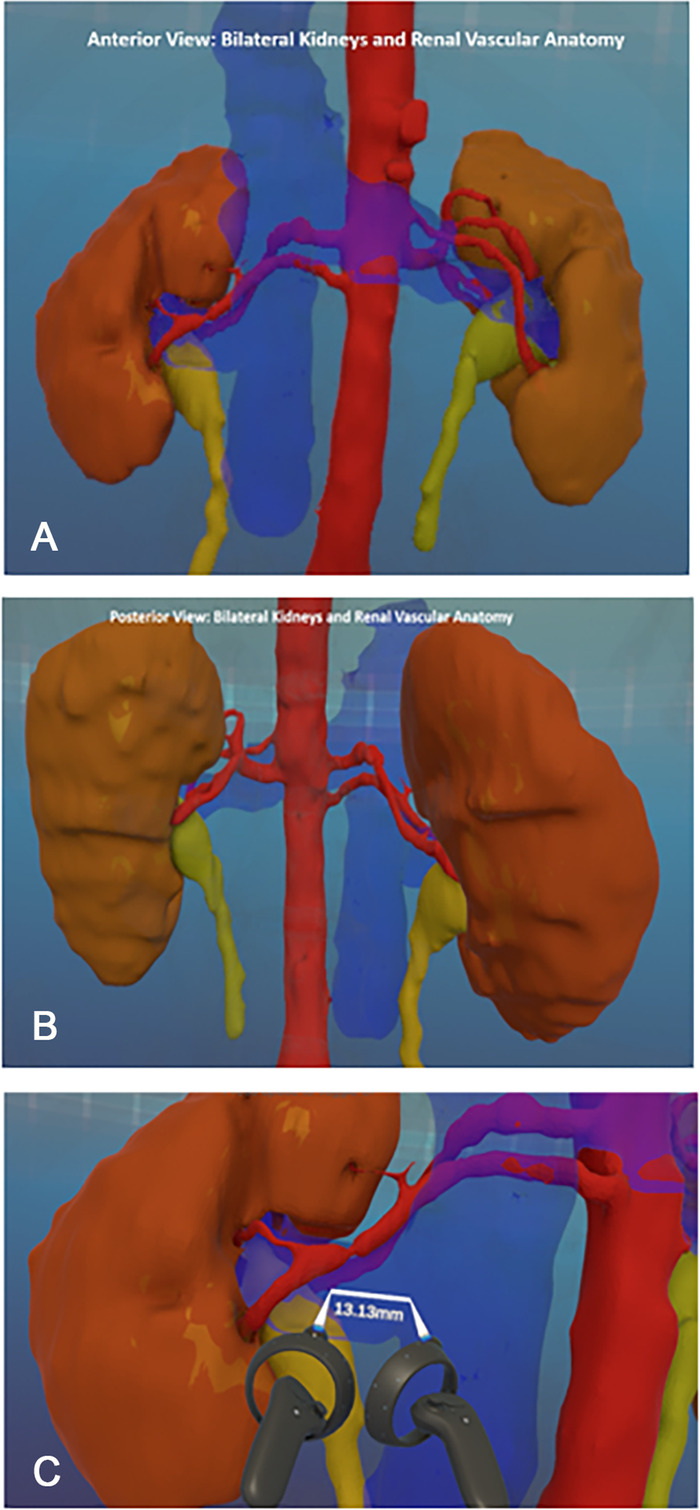
**Arterial anatomy is highlighted in red in the (A) anterior view and (B) posterior view of the bilateral kidneys from the 3-dimensional reconstruction. (C) Handsets that accompany the headset can be used to zoom in on pertinent structures and make measurements in the immersive virtual reality environment.** (For readers of the print publication, a color version of this figure is available at https://doi.org/10.31486/toj.22.0008.)

The left kidney, however, had a normal vein and 2 arteries, each bifurcating at an acceptable distance from the aorta. The points of bifurcation and crossing were clearly defined, favoring plenty of room for safe vascular stapler placement during the recovery operation. Static images were captured and shared with the multidisciplinary team at the selection conference (Figure 4). Considering this new information, the donor was re-presented to the selection committee and was approved for donation of the left kidney.

The donor was appropriately consented for donation, with extra emphasis placed on the possibility that the multiple vessels might require complex reconstruction and the potential necessity of conversion to open surgery to safely recover the kidney. The donor's scans were reviewed with standard 2D modalities as well as in VR prior to his operation. He underwent left laparoscopic, robot-assisted donor nephrectomy and left the hospital on the first postoperative day. In surgery, the donor's anatomy was found to be as expected from the 3D VR visualizations. Two renal arteries fed the left kidney, with each bifurcated far enough away from the aorta to be safely stapled and divided.

The recipient underwent a successful transplant, after side-to-side/pair-of-pants–type renal artery reconstruction of the 2 left arteries on the backbench. At 16 months posttransplant, the recipient maintained good graft function, despite recurrent FSGS that required treatment. He has had no ureteral or vascular complications.

## DISCUSSION

Application of technology to living donor nephrectomy is not new, with the goal of improving the donor's safety and recovery from a highly technical yet medically unnecessary procedure. Because of the widespread proficiency in laparoscopy, minimally invasive donor nephrectomy has become the standard.^7^ Advances in medical imaging and visualization allow for detailed understanding of discrete donor anatomy as well as planning of the donor and recipient operations. Still, on occasion, a willing donor is declined because of prohibitive anatomy demonstrated on multiphase contrast-enhanced CT.^8^

Thin-slice multiphase contrast-enhanced CT is critical in assessing donor anatomy, but its interpretation becomes increasingly difficult as renal anatomy becomes more complex. Up to 30% of potential donors have more than 1 renal artery for a kidney.^9-11^ In a comparison of CTA vascular anatomy and intraoperative findings in 2,144 living kidney donors, CTA identified the correct number of renal arteries in 97.9% of cases.^10^ While none of the patients had more arteries than predicted, 2.1% had fewer arteries found intraoperatively than were demonstrated on CTA.^10^ Thus, CTA can lead to a misinformed decision to turn down a potential living kidney donor on the basis of complex vascular anatomy as was the case with our donor.

Regarding volume measurements, 3D reconstruction and VR viewing allow for more precise volume measurements than 2D imaging can reliably provide.^12^ With traditional 2D imaging, length, width, and depth of each kidney may be measured and multiplied to approximate the kidney's gross volume. Nuclear medicine imaging can be performed to measure the split function of each kidney but requires additional costly or inconvenient testing. Additionally, the results of nuclear medicine split function testing have been shown to be similar to and possibly inferior to CT volumetry at predicting postdonation glomerular filtration rate.^13^

Surgeons generally report that they use axial, coronal, and sagittal 2D images to build a 3D model of the anatomy in their mind. Surgeons’ skill at this mental reconstruction may not be as accurate as they believe, however, as cognitive localization of renal tumors based on 2D images has been shown to be poor, and it improves when 3D modeling is provided.^14^ When presented with 3D models of patient anatomy, surgeons rate these as highly useful clinical tools and techniques.^15^ Further, a 2021 study in the live kidney donor population found interactive VR highly valuable, improving surgeons’ understanding of anatomy, decreasing preoperative anxiety, and decreasing operative time.^16^

For this case, we chose VR because of its immersive nature and stereoscopic environment. Other modalities of 3D visualization without VR or 3D printing are available, including some used by radiology departments such as Synapse 3D (Fujifilm Corporation) and software-as-service offerings such as Ceevra. Access to these programs varies by service line and by institution. These software products were not available to the transplant team when the donor's CTA images were evaluated.

Regarding the cost of the technology used in our case, while de novo startup costs could be high—approximately $100,000 for a biomedical engineer, $15,000 for US Food and Drug Administration–approved 3D segmentation software, and $5,000 for a capable computer and display hardware—many of these assets are already in place in hospital systems and universities. In our case, we developed relationships with in-house teams already using this technology, and our cost was only $250 for the time the biomedical engineer needed to create the model.

Recipient outcomes following transplantation of a donor kidney with 2 renal arteries have been demonstrated to be acceptable compared to outcomes after transplantation of a donor kidney with a single renal artery, and reconstruction techniques are typically straightforward.^5,17^

Donor kidneys with more than 2 arteries have been shown to have some inferior outcomes compared to those with 2 or 1, namely longer cold times, increased delayed graft function, and increased slow graft function, but equivalent long-term graft survival.^18^ Also, the additional vascular complexity increases the risk for converting to open surgery during the donor nephrectomy, putting the donor at higher risk.^11^

Liberal use of 3D reconstruction and VR viewing may elucidate additional clinical scenarios in which 3D visualization is warranted and identify precisely which conditions, pathologies, or organ systems are most amenable to 3D VR visualization. We have begun to use 3D reconstruction at our institution routinely for the kidney donor population, which has led our team to identify extrarenal structures, such as ribs, spine, and liver or spleen, as useful reference anatomy in relation to the renal vascular anatomy. In recipients, 3D visualization could be used to clarify iliac calcifications or to better appreciate the intra-abdominal space occupied by polycystic kidneys.^19^ With continued routine use of 3D reconstruction, the entire team (surgeons, nephrologists, nurses, other staff) will grow familiar with the technology and how it can be applied.

**Figure 5.**
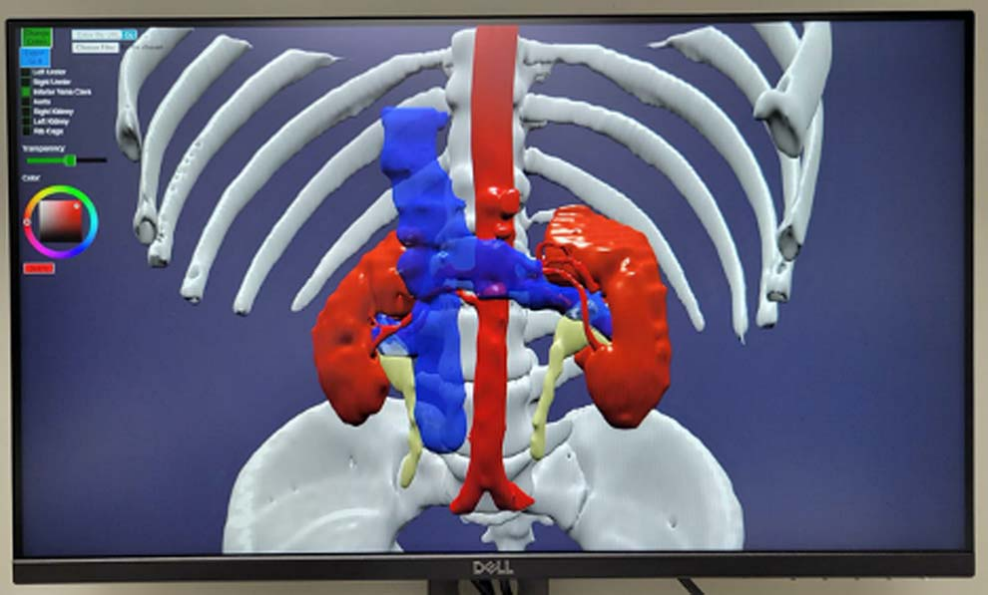
Three-dimensional models can be explored on a desktop monitor without requiring a headset.

The VR headset is cumbersome for some users, so a more convenient way of visualizing the 3D model was needed. Creation of a desktop/laptop screen viewer for the 3D models allows for increased accessibility by not requiring the headset but does sacrifice the immersive nature of VR (Figure 5). Nonsurgeon team members are not often well versed in reading CT anatomy, but with a computer screen viewer at selection committee, all attendees can see anatomic details referenced by the CT scan. Alternatively, holographic displays preserve stereoscopy for multiple users without the need for headsets. Our institution is developing an electronic medical record order to conveniently and systematically request and track utilization of 3D visualization in LDRT.

3D printing of models segmented from patient CT images is useful in select cases. 3D prints allow for improved communication regarding surgical planning and operative technique, as well as the opportunity for improved patient education, comprehension, and satisfaction.^20,21^

## CONCLUSION

The use of 3D reconstruction with VR visualization to clarify complex renal vascular anatomy allowed a living kidney donor to donate a kidney to his brother after initially being declined by the selection committee. While these technologies may not be necessary for routine LDRT, they were instrumental in this case and should be considered in cases requiring discrete appreciation of complex anatomy or in cases with an anatomic feature that is possibly a contraindication to donation.

## Notes

*This article meets the Accreditation Council for Graduate Medical Education and the American Board of Medical Specialties Maintenance of Certification competencies for Patient Care and Medical Knowledge.*
